# TREC/KREC Levels and T and B Lymphocyte Subpopulations in COVID-19 Patients at Different Stages of the Disease

**DOI:** 10.3390/v14030646

**Published:** 2022-03-21

**Authors:** Andrei A. Savchenko, Elena Tikhonova, Igor Kudryavtsev, Dmitry Kudlay, Ilya Korsunsky, Vasily Beleniuk, Alexandr Borisov

**Affiliations:** 1 Federal Research Center “Krasnoyarsk Science Center of the Siberian Branch of the Russian Academy of Sciences”, Scientific Research Institute of Medical Problems of the North, 660022 Krasnoyarsk, Russia; aasavchenko@yandex.ru (A.A.S.); dyh.88@mail.ru (V.B.); 2410454@mail.ru (A.B.); 2 Ministry of Health of the Russian Federation, V.F. Voino-Yasenetsky Krasnoyarsk State Medical University, 660022 Krasnoyarsk, Russia; tihonovaep@mail.ru; 3 Institute of Experimental Medicine, 197376 St. Petersburg, Russia; 4 Institute of Life Sciences and Biomedicine, Far Eastern Federal University, 690922 Vladivostok, Russia; 5 National Research Center—Institute of Immunology, Federal Medical-Biological Agency, 115522 Moscow, Russia; d624254@gmail.com; 6 Ministry of Health of the Russian Federation, I.M. Sechenov First Moscow State Medical University (Sechenov University), 119991 Moscow, Russia; 7 Moscow City Center for Pediatric Immunology and Allergy, G. Speransky Children’s Hospital No 9, 129329 Moscow, Russia; iliakors@gmail.com

**Keywords:** COVID-19 patients, T lymphocyte, B lymphocyte, TREC, KREC

## Abstract

Background: T and B cell-mediated immunity can be assessed using T cell receptor excision circle (TREC) and Kappa-deleting recombination excision circle (KREC) analysis, respectively, and successful implementation of this method requires evaluation of the correlation between the TREC frequencies and T cell subsets as well as KREC levels and B lymphocyte subsets. The aim of the present study was to evaluate the correlation between the TREC/KREC concentrations and T/B lymphocyte subsets at different stages of COVID-19. Methods: We examined 33 patients in the acute stage of COVID-19 (including 8 patients with poor outcomes) and 33 COVID-19 survivors. TREC/KREC concentrations were measured using quantitative real-time PCR. T/B lymphocyte subsets were determined using flow cytometry. Results: Blood TREC and KREC levels were found to be significantly lower in the acute stage of COVID-19 compared to control values. Moreover, a zero blood TREC level was a predictor of a poor disease outcome. Reductions in CD3^+^CD4^+^CD45RO^−^CD62L^−^ and CD3^+^CD8^+^CD45RO^−^CD62L^−^ T cell counts (as well as in the main fractions of B1 and B2 B cells) indicated a favorable outcome in COVID-19 patients in the acute stage of the disease. Decreased CD3^+^CD4^+^CD45RO^−^CD62L^+^ and CD3^+^CD8^+^CD45RO^−^CD62L^+^ T cell frequencies and increased CD3^+^CD8^+^CD45RO^−^CD62L^−^ cell counts were found to indicate a poor outcome in patients with acute COVID-19. These patients were also found to have increased B1 cell counts while demonstrating no changes in B2 cell counts. The levels of effector T cell subsets an naïve B cells were normal in COVID-19 survivors. The most pronounced correlations between TREC/KREC levels and T/B cell subsets counts were observed in COVID-19 survivors: there were positive correlations with naïve T and B lymphocytes and negative correlations with central and effector memory T cell subsets. Conclusions: The assessment of correlations between TREC and T cell subsets as well as KREC levels and B cell subset counts in patients with acute COVID-19 and COVID-19 survivors has shown that blood concentrations of TREC and KREC are sensitive indicators of the stage of antigen-independent differentiation of adaptive immunity cells. The results of the TREC and KREC analysis correlated with the stages of COVID-19 and differed depending on the outcome of COVID-19.

## 1. Introduction

The effectiveness of the immune response in patients with any infection is largely determined by the functional activity of T and B lymphocytes. In the process of respiratory transfer, SARS-CoV-2 infects the alveolar epithelial cells, endothelial cells of blood vessels and macrophages and causes the reduction or complete cessation of interferon synthesis [1,2,3]. Therefore, there is no interferon-dependent inhibition of viral replication, no migration of viral-specific T lymphocytes and no synthesis of neutralizing antibodies in the site of inflammation. In case of disorders of a local protective immune response and/or adaptive immunity, the cytokine synthesis increases substantially, resulting in the development of systemic inflammatory response syndrome (hypercytokinemia or the cytokine storm). The cytokine storm results in the involvement of organs and systems and the development of acute multiorgan failure [4,5,6,7].

Blood T and B cell counts were shown to decrease during the cytokine storm [8,9]. CD4^+^ and CD8^+^ T cell frequencies were inversely proportional to IL-6 and IL-10 concentrations, which increased with the severity of SARS-CoV-2 infection [10]. At the same time, an analysis of peripheral blood T helper cell differentiation showed that COVID-19 patients had lower proportions of CD4+ T cells with the CD45RA^+^CD27^+^CCR7^+^CD95^−^ phenotype, while the counts of EM2 and EMRA (with CD45RA^−^CD27^−^CCR7^+^ and CD45RA^+^CD27^−^CCR7^−^ phenotypes) cells were found to be significantly increased compared to control values [11]. Peripheral blood CD19^+^ B cell levels in patients with COVID-19 during the acute stage of the disease were lower compared to control values; however, there was an increase in the proportion of circulating CD27^hi^CD38^hi^CD24^−^ plasma cell precursors [11].

Currently, there are many original research studies analyzing the profile of adaptive immunity cells in COVID-19 survivors. COVID-19 convalescents were found to have increased concentrations of IL-17 and counts of TNFα-producing CD4^+^ T helpers, while both the TNFα^+^ and IFNγ^+^ cell percentages were increased among cytotoxic T cells [12]. In general, the alterations in T and B lymphocyte maturation and differentiation in COVID-19 patients in the acute stage of the disease and COVID-19 survivors were assumed to be related to the functions of the central and peripheral organs of the immune system.

A method for the assessment of T and B cell-mediated immunity based on the determination of the content of T cell receptor excision circles (TREC) and Kappa-deleting recombination excision circles (KREC) is currently being implemented [13,14,15]. TREC are extrachromosomal circular excision products of the rearrangement of T cell receptor (TCR) genes during somatic DNA recombination, which are formed during the maturation of T cells in the thymus. Circular TREC molecules are formed in double-negative thymocytes (DN4 thymocytes) at the stage of rearrangement of the TCR α-chain upon recombination of the δRec and ψJα elements, followed by deletion of the TCRδ locus. TREC are detected in thymocytes and naïve T lymphocytes. KREC is formed by a similar mechanism at the stage of differentiation of pre-B cells and V(D)J-recombination of immunoglobulin light chain genes [16,17,18]. Thus, the amount of TREC and KREC in the blood characterizes the level of differentiation of T and B cells in the central organs of the immune system. The TREC/KREC assay has been successfully used in pediatric patients with primary immunodeficiencies [16,17,18]. A few studies have demonstrated the significance of the determination of TREC and KREC levels for the analysis of immunity in patients with various infectious diseases. A study by Khadzhieva M.B. et al. (2021) provided the results of the TREC/KREC assay in COVID-19 patients [14]. The results of this test could be used not only to assess the state of adaptive immunity but also to develop new methods of prognosis of the disease course and the outcomes of a wide range of infectious diseases, including COVID-19.

The aim of this study was to determine the correlation between the TREC and KREC concentrations and the T and B lymphocyte subset levels in COVID-19 patients at different stages of the disease.

## 2. Materials and Methods

### 2.1. Patients and Study Design

A total of 33 COVID-19 patients in the acute stage of the disease, 33 COVID-19 survivors, and 24 virtually healthy patients (control group) were enrolled in a pilot controlled open-label non-randomized experimental clinical study conducted at the Scientific Research Institute of Medical Problems of the North (Federal Research Center “Krasnoyarsk Science Center” of the Siberian Branch of the Russian Academy of Sciences) and the Regional State Budgetary Healthcare Institution “Krasnoyarsk Interregional Clinical Emergency Medicine Hospital named after N.S. Karpovich”). The criteria for clinical symptoms of COVID-19 patients were the following: moderate or severe body temperature above 38 °C for more than 3 days, dyspnea (with at least 1 respiratory (catarrhal) symptom—cough, sore throat, chest congestion, symptoms of rhinitis, pharyngitis, tracheitis), SpO2 < 95%, and at least 1 symptom of intoxication (headache, malaise, myalgia, sweating and/or chills, weakness). Persistent febrile fever, acute respiratory distress syndrome, acute respiratory failure with the need for respiratory support, septic shock, multiple organ failure, and changes in the lungs on computed tomography were diagnosed in extremely severe cases in COVID-19 patients.

All study subjects signed the informed consent form, and the study was approved by the Ethics Committees of the Scientific Research Institute of Medical Problems of the North (Federal Research Center “Krasnoyarsk Science Center” of the Siberian Branch of the Russian Academy of Sciences) and Regional State Budgetary Healthcare Institution “Krasnoyarsk Interregional Clinical Emergency Medicine Hospital named after N.S. Karpovich”) in accordance with the Declaration of Helsinki of the World Medical Association “Ethical Principles for Medical Research Involving Human Subjects” (amended in 2013), and the “Rules for Good Clinical Practice in the Russian Federation” approved by Decree No. 266 of the Ministry of Health of the Russian Federation of 19 June 2003.

**Figure 1:**
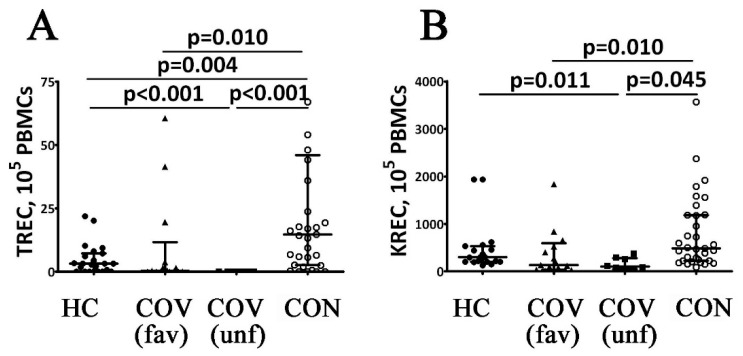
T cell receptor excision circles (TREC) and Kappa-deleting recombination excision circles (KREC) counts in patients with acute COVID-19 and COVID-19 convalescent patients. Scatters plots (**A**,**B**) showing the numbers (copies per 105 cells) of TRECs and KRECs, respectively Black circles—healthy control (HC, *n* = 24); black triangles—patients with acute COVID-19 (COV (fav), favorable outcome, *n* = 25); black squares—patients with acute COVID-19 (COV (unf), unfavorable outcome, *n* = 8); and white circles—COVID-19 survivors (CON, convalescent patients with favorable outcome of acute COVID-19, *n* = 33). Each dot represents individual subjects, and horizontal bars depict the group medians and quartile ranges (Med (Q25; Q75). Statistical analysis was performed with the Mann–Whitney U test.

**Figure 2:**
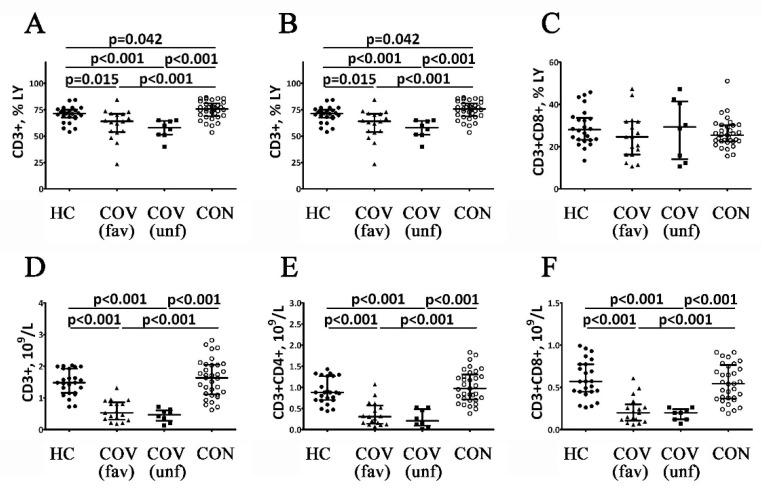
Altered relative and absolute numbers of main T cell subsets in patients with acute COVID-19 and COVID-19 convalescent patients. Scatters plots (**A**–**C**) and (**D**–**F**) showing the percentages (the percentage of T cell subset within total lymphocyte population) and absolute numbers (number of cells in 1 liter of peripheral blood, 10^9^/L) of T cells (CD3^+^), T-helpers (CD3^+^CD4^+^) and cytotoxic T cells (CD3^+^CD8^+^), respectively. Black circles—healthy control (HC, *n* = 24); black triangles—patients with acute COVID-19 (COV (fav), favorable outcome, *n* = 25); black squares—patients with acute COVID-19 (COV (unf), unfavorable outcome, *n* = 8); and white circles—COVID-19 survivors (CON, convalescent patients with favorable outcome of acute COVID-19, *n* = 33). Each dot represents individual subjects, and horizontal bars depict the group medians and quartile ranges (Med (Q25; Q75). Statistical analysis was performed with the Mann–Whitney U test.

**Figure 3:**
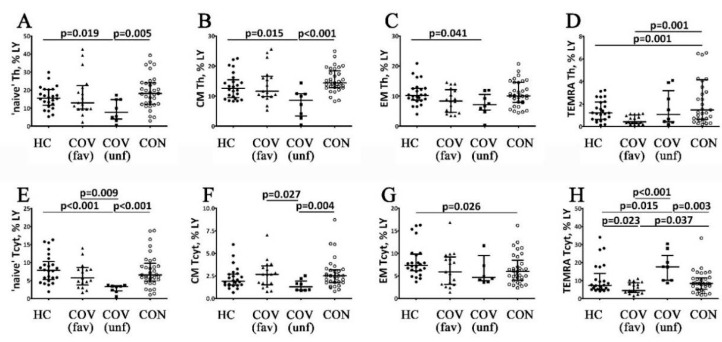
Alterations in relative numbers of T cell subsets with different patterns of CD45RO and CD62L expression in patients with acute COVID-19 and COVID-19 convalescent patients. Scatters plots (**A**–**D**) and (**E**–**H**) showing the percentages of ‘naïve’ (CD45RO^−^CD62L^+^), central memory (CM, CD45RO^+^CD62L^+^), effector memory (EM, CD45RO^+^CD62L^−^) and terminally differentiated CD45RA-positive effector memory (TEMRA, CD45RO^−^CD62L^−^) T-helpers and cytotoxic T cells, respectively. The data represents the percentages of T cell subsets within total lymphocyte population. Black circles—healthy control (HC, *n* = 24); black triangles—patients with acute COVID-19 (COV (fav), favorable outcome, *n* = 25); black squares—patients with acute COVID-19 (COV (unf), unfavorable outcome, *n* = 8); and white circles—COVID-19 survivors (CON, convalescent patients with favorable outcome of acute COVID-19, *n* = 33). Each dot represents individual subjects, and horizontal bars depict the group medians and quartile ranges (Med (Q25; Q75). Statistical analysis was performed with the Mann–Whitney U test.

**Figure 4:**
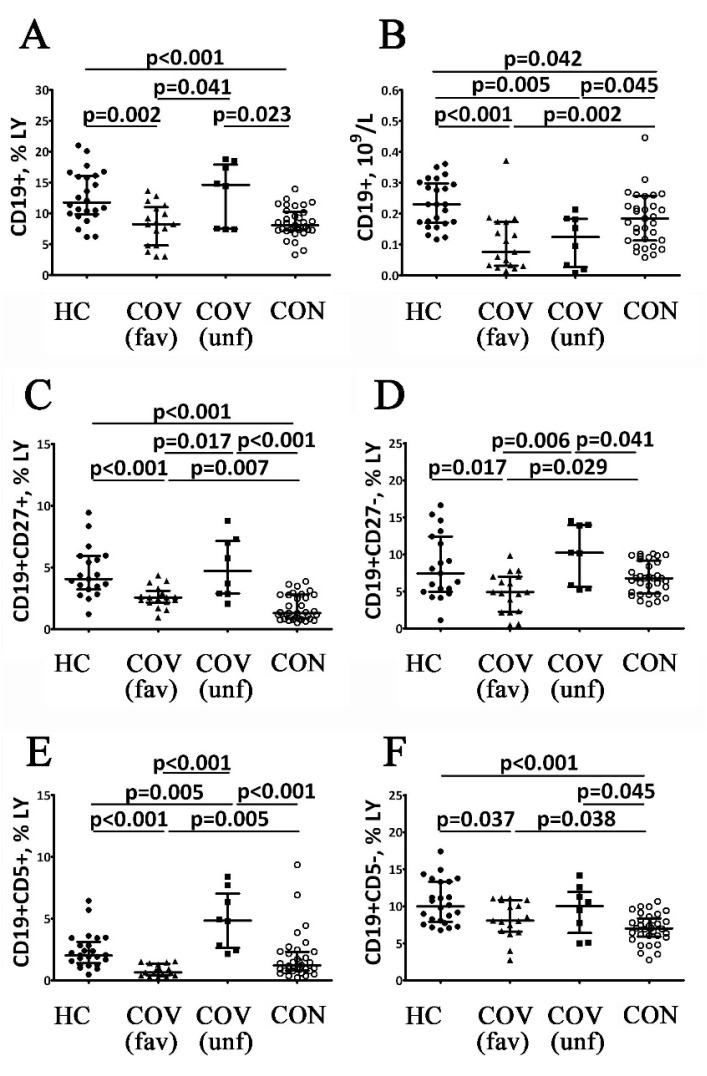
Alterations in B cell subsets in patients with acute COVID-19 and COVID-19 convalescent patients. Scatter plots (**A**,**B**) show the percentages (the percentage of B cells within total lymphocyte population) and absolute numbers (number of B cells in 1 L of peripheral blood, 10^9^/L) of B lymphocytes (CD19+), respectively. Scatter plots (**C**,**D**) show the percentages (the percentage of B cell subsets within the total lymphocyte population) of CD27-positive and CD27-negative B cells, respectively. Scatter plots (**E**,**F**) show the percentages (the percentage of B cell subsets within total lymphocyte population) of CD5-positive and CD5-negative B cells, respectively. Black circles—healthy control (HC, *n* = 24); black triangles—patients with acute COVID-19 (COV (fav), favorable outcome, *n* = 25); black squares—patients with acute COVID-19 (COV (unf), unfavorable outcome, *n* = 8); and white circles—COVID-19 survivors (CON, convalescent patients with favorable outcome of acute COVID-19, *n* = 33). Each dot represents individual subjects, and horizontal bars depict the group medians and quartile ranges (Med (Q25; Q75). Statistical analysis was performed with the Mann–Whitney U test.

**Figure 5:**
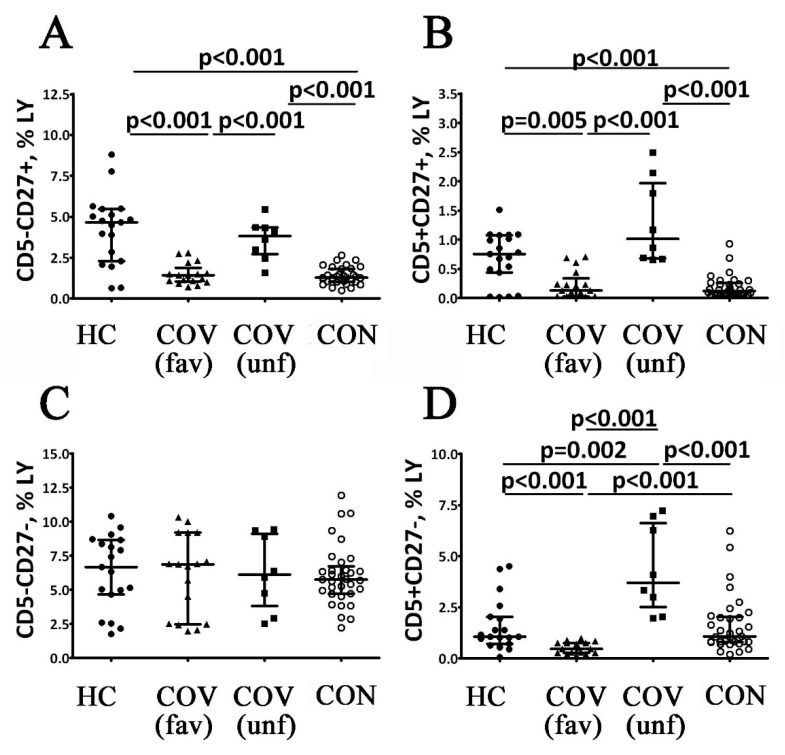
Alterations in relative numbers of B cell subsets with different patters of CD5 and CD27 expression in patients with acute COVID-19 and COVID-19 convalescent patients. Scatter plots (**A**–**D**) show the percentages of CD5^−^CD27^+^, CD5^+^CD27^+^, CD5^−^CD27^−^ and CD5^+^CD27^−^ B cells, respectively. The data represents the percentages of B cell subsets within the total lymphocyte population. Black circles—healthy control (HC, *n* = 24); black triangles—patients with acute COVID-19 (COV (fav), favorable outcome, *n* = 25); black squares—patients with acute COVID-19 (COV (unf), unfavorable outcome, *n* = 8); and white circles—COVID-19 survivors (CON, convalescent patients with favorable outcome of acute COVID-19, *n* = 33). Each dot represents individual subjects, and horizontal bars depict the group medians and quartile ranges (Med (Q25; Q75). Statistical analysis was performed with the Mann–Whitney U test.

Blood sampling in patients with acute COVID-19 was carried out within the first 24 h after diagnosis. Depending on the outcome of the acute stage of the disease, the patients were divided into 2 groups: acute stage of COVID-19 with a favorable outcome (25 patients, 14 females and 11 males, marked as “COV (fav)” on Figures 1–5) and acute stage of COVID-19 with a poor outcome (8 patients, 6 females and 2 males, marked as “COV (unf)” on Figures 1–5). The diagnosis, treatment, and evaluation of COVID-19 were based on the Temporary Methodical Guidelines developed by the Ministry of Health of the Russian Federation (version 11 of 7 May 2021) [19]. COVID-19 convalescent patients or COVID-19 survivors (18 females and 15 males, marked as “CON” on Figures 1–5) were examined 3 months after the discharge from the Infectious Disease Unit of Regional State Budgetary Healthcare Institution “Krasnoyarsk Interregional Clinical Emergency Medicine Hospital named after N.S. Karpovich.” The mean age of the examined patients was 62 years (57–70 years). All study groups were comparable regarding age and gender. Clinical characteristics of the groups are presented in Table 1.

### 2.2. Determination of TREC and KREC Concentrations

TREC and KREC DNA concentrations were determined by quantitative real-time PCR using Immuno-BiT test kit (ABV-TEST LLC, Moscow, Russia) and LightCycler 96 (Roche, Munchen, Germany) and Rotor-Gene 6000 (Corbett Research, Sydney, Australia) amplifiers according to the manufacturer’s instructions. The normalized TREC and KREC values were estimated for 10^5^ leukocytes, taking into account the serum albumin (ALB)-based internal control by the method suggested by M.A. Gordukova et al. (2019) [16].

### 2.3. Evaluation of T and B cell Subsets by Flow Cytometry

For the immunophenotyping of T cell subsets, each whole peripheral blood sample (100 μL) was stained using FITC-labeled mouse anti-human CD62L (clone DREG56, cat. IM1231U, Beckman Coulter, INpolis, IN, USA), PE-labeled mouse anti-human CD8 (clone B9.11, cat. A07757, Beckman Coulter, INpolis, IN, USA), ECD-labeled mouse anti-human CD45R0 (clone UCHL1, cat. B49192, Beckman Coulter, INpolis, IN, USA), PC7-labelled mouse anti-human CD4 (clone T4, cat. 6607101, Beckman Coulter, INpolis, IN, USA), PE-labelled mouse anti-human CD8 (clone B9.11, cat. A07757, Beckman Coulter, INpolis, IN, USA), A-A700-labelled mouse anti-human CD3 (clone UCHT1, cat. B10823, Beckman Coulter, INpolis, IN, USA) and A-A750-labelled mouse anti-human CD45 (clone J33, cat. A79392, Beckman Coulter, INpolis, IN, USA). For B cell subsets immunophenotyping, we used the following antibodies: FITC-labeled mouse anti-human CD19 (clone J3-119, cat. A07768, Beckman Coulter, INpolis, IN, USA), PE-labeled mouse anti-human CD5 (clone BL1a, cat. A07753, Beckman Coulter, INpolis, IN, USA), ECD-labeled mouse anti-human CD27 (clone 1A4CD27, cat. B26603, Beckman Coulter, INpolis, IN, USA) and A-A750-labelled mouse anti-human CD45 (clone J33, cat. A79392, Beckman Coulter, INpolis, IN, USA). The staining protocols were performed according to the manufacturer’s recommendations. The distribution of antibodies by fluorescence channels was determined in accordance with the principles of panel formation for multicolor flow cytometry tests [20]. Sample preparation was carried out using the conventional technique [21]. The stained cells were examined using a Navios flow cytometer (Beckman Coulter, INpolis, IN, USA) of the Center for Collective Use of the “Krasnoyarsk Science Center” of the Siberian Branch of the Russian Academy of Sciences. The flow cytometry results were analyzed using Navios Software v. 1.2 and Kaluza v. 2.1.1 (Beckman Coulter, INpolis, IN, USA). At least 50,000 lymphocytes were analyzed in each sample. 

### 2.4. Statistical Data Analysis

The sample was described by calculating the median (Me) and the interquartile range in the form of Q1 and Q3 (C_25_–C_75_). The significance of differences between independent samples was assessed using the non-parametric Mann–Whitney U test. The Spearman rank R was used to study the correlation between the parameters. Statistical data analysis was carried out using Statistica 8.0 (StatSoft, Tulsa, OK, USA; 2007) applied software.

## 3. Results

### 3.1. Determination of TREC and KREK Levels in COVID-19 Patients at Different Stages of the Disease

TREC and KREC DNA levels in COVID-19 patients were studied by real-time PCR. The patients in the acute stage of the disease with a favorable outcome were found to have a significant decrease in TREC and KREC levels compared to control values (see Figure 1). Moreover, COVID-19 patients with poor outcomes demonstrated zero blood levels of TREC DNA during the acute stage of the disease, which determined statistically significant differences not only with the control group results but also with those in COVID-19 patients with a favorable outcome of the acute stage of the disease. At the same time, KREC DNA levels in patients with poor outcomes were similar to those observed in patients with favorable outcomes of the acute stage of COVID-19. However, they were significantly lower compared to the control group. Finally, blood concentrations of TREC and KREC in COVID-19 survivors were increased up to the reference range and became significantly higher than the values during the acute stage of the disease.

### 3.2. T cell Subsets in COVID-19 Patients at Different Stages of the Disease

The results of the assessment of T cell subsets in COVID-19 patients at different stages of the disease are shown in Figure 2. Firstly, COVID-19 patients with favorable outcomes of the acute stage of the disease showed decreased percentages and absolute values of the CD3^+^ cells. In patients with poor disease outcomes, the decrease of the CD3^+^ cell percentages was even more pronounced (relative to the reference range and the levels observed in patients with favorable outcomes), while their absolute numbers were decreased only if compared to control (Figure 2A). COVID-19 survivors demonstrated higher CD3^+^ cell percentages compared to the control group and both patient groups in the acute stage of the disease. The absolute numbers of CD3^+^ cells in COVID-19 survivors were within the reference ranges but higher than in patients in the acute stage of the disease (Figure 2D).

COVID-19 patients with favorable outcomes had lower absolute CD4^+^ T cell counts during the acute stage of the disease compared to the control values (Figure 2B). Absolute and related numbers of CD4^+^ T cell were decreased in patients with poor outcomes of the acute stage of the disease (Figure 2E). COVID-19 survivors demonstrated higher percentages of Th cells compared to control values and patients with acute COVID-19. Furthermore, the absolute Th counts in COVID-19 survivors were within the reference range but higher than in patients during the acute stage of the disease. Moreover, the percentages of CD45RO^−^CD62L^−^ Th cells in COVID-19 patients with favorable outcomes decreased compared with control values during the acute stage of COVID-19 (Figure 3D). COVID-19 patients with poor outcomes had decreased levels of CD45RO^−^CD62L^+^ and CD45RO^+^CD62L^+^ Th cell during the acute stage compared to the reference ranges and the values observed in patients with favorable disease outcomes (Figure 3A,B). Moreover, CD45RO^+^CD62L^−^ Th cell percentages in COVID-19 patients with poor outcomes were decreased only if compared to controls, while the CD45RO^−^CD62L^−^ Th cell frequency in this group was higher compared to the patients with favorable disease outcomes (Figure 3C,D). COVID-19 survivors had higher percentages of CD45RO^−^CD62L^+^, CD45RO^+^CD62L^+^ and CD45RO^+^CD62L^−^ Th cells compared to the values observed in patients with poor outcomes in the acute stage of the disease (Figure 3B,C). However, CD45RO^−^CD62L^−^ Th cell levels in COVID-19 survivors were higher than in patients with favorable outcomes in the acute stage of the disease (Figure 3D).

The absolute numbers of peripheral blood CD3^+^CD8^+^ cells (Tcyt) in COVID-19 patients during the acute stage of the disease were similar regardless of the disease outcome but were lower compared to healthy controls (Figure 1C,F). Furthermore, CD45RO^−^CD62L^−^ Tcyt percentages in COVID-19 patients with a favorable outcome in the acute stage of the disease were decreased compared to control values (Figure 2H). At the same time, COVID-19 patients with a poor outcome in the acute stage of the disease exhibited lower CD45RO^−^CD62L^+^ Tcyt and higher CD45RO^−^CD62L^−^ Tcyt frequencies compared to the control group and COVID-19 patients with a favorable disease outcome (Figure 2E,H). Next, CD45RO^+^CD62L^+^ Tcyt levels in COVID-19 patients with a poor disease outcome decreased compared to the patients with a favorable disease outcome (Figure 3F). Blood CD3^+^CD8^+^ cell counts in COVID-19 survivors were within the reference range but higher than in patients during the acute stage of the disease. Interestingly, CD45RO^−^CD62L^+^- and CD45RO^+^CD62L^+^ Tcyt frequencies in COVID-19 survivors were significantly higher than in the patients with a poor outcome in the acute stage of the disease (Figure 2E,F). Finally, in this group, CD45RO^+^CD62L^−^ Tcyt numbers were lower compared to the control values, while CD45RO^−^CD62L^−^ Tcyt levels were increased compared to the patients with a favorable disease outcome but decreased compared to COVID-19 patients with a poor outcome in the acute stage of the disease (Figure 3G,H).

### 3.3. B cell Subsets in COVID-19 Patients at Different Stages of the Disease

The results of an assessment of B cell subsets in COVID-19 patients at different stages of the disease are shown in Figure 4 and Figure 5. COVID-19 patients with a favorable outcome of the acute stage of the disease showed decreased relative and absolute numbers of the CD19^+^ cells (Figure 4A,B). This decrease in B cells were closely linked with a decrease of CD19^+^CD27^+^ and CD19^+^CD27^−^ B cells (Figure 4C,D). Total CD19^+^ cell frequencies in COVID-19 patients with a poor outcome in the acute stage of the disease showed no differences with reference ranges but were significantly higher than in patients with a favorable outcome (Figure 3A,B). Furthermore, the relative cell numbers were lower when compared to the reference range. Moreover, COVID-19 patients with poor disease outcomes had higher percentages of CD19^+^CD27^−^ and CD19^+^CD27^+^ B cells in circulation than patients with favorable outcomes (Figure 4C,D). COVID-19 survivors had lower percentages of CD19^+^ cells compared to reference ranges and the levels exhibited by patients with poor outcomes. The absolute CD19^+^ cell counts in these patients were also lower than healthy controls and patients with a favorable COVID-19 outcome (Figure 4A,B). Furthermore, COVID-19 survivors demonstrated decreased circulating CD19^+^CD27^−^ B cells compared to patients with a poor outcome in the acute stage of the disease, while the CD19^+^CD27^+^ B cell numbers in this group were lower compared to controls or patients with a poor outcome (Figure 4C,D).

COVID-19 patients with a favorable outcome in the acute stage of the disease had lower levels of CD5^+^ and CD5^−^ cells compared to the control values (Figure 4). This group had lower CD5^+^ B cell numbers and lower CD19^+^CD5^+^CD27^−^ and CD19^+^CD5^+^CD27^+^ cell counts compared to healthy controls (Figure 5). Moreover, the patients with a favorable disease outcome also had lower CD19^+^CD5^−^ cell counts, while only CD19^+^CD5^+^CD27^+^ cells were down-regulated (Figure 4F and Figure 5B). COVID-19 patients with a poor outcome in the acute stage of the disease demonstrated higher percentages of CD19^+^CD5^+^ and CD19^+^CD5^−^ cells in relation to both the control values and the values observed in patients with a favorable disease outcome (Figure 4E,F). This patient group had higher CD19^+^CD5^+^CD27^−^ and CD19^+^CD5^+^CD27^+^ cell counts compared with reference ranges and patients with a favorable disease outcome (Figure 5B,D). However, COVID-19 patients with a poor disease outcome demonstrated higher CD19^+^CD5^+^CD27^+^ cell levels compared to patients with a favorable disease outcome (Figure 5B). In COVID-19 survivors, the frequencies of CD5^+^ B cells demonstrated a two-fold increase compared to patients with a favorable outcome in the acute stage of the disease but were significantly lower when compared to patients with a poor disease outcome (Figure 4E). CD19^+^CD5^+^CD27^−^ cell frequencies in this group were also higher than in patients with a favorable disease outcome but lower than in patients with a poor disease outcome (Figure 5D). However, CD19^+^CD5^+^CD27^+^ cell counts were lower than the control group and the patients with poor disease outcomes (Figure 5B). Finally, COVID-19 survivors had lower CD5^−^ and CD5^−^CD27^+^ B cell counts than the control group and patients with a poor disease outcome (Figure 4F and Figure 5A). Differences between the examined groups of patients were completely absent in the number of CD19^+^CD5^−^CD27^−^ cells (Figure 5C).

### 3.4. Correlation of TREC and KREC Values with T and B Cell Subsets at Different Stages of COVID-19

A correlation analysis was applied to evaluate relationships between the TREC and KREC levels and T and B cell subsets in the control group and COVID-19 patients at different stages of the disease. The control group demonstrated the absence of correlations between these parameters. However, in COVID-19 patients with a favorable outcome in the acute stage of the disease, there was a positive correlation between the KREC levels and the frequencies of CD19^+^CD5^+^ (r = 0.54, *p* = 0.025) and CD19^+^CD5^−^ cells (r = 0.49, *p* = 0.048). COVID-19 patients with a poor disease outcome in the acute stage of the disease demonstrated no correlations.

In COVID-19 survivors, TREC levels demonstrated a negative correlation with naïve CD45RO^+^CD62L^+^ Th cells (r = −0.56, *p* = 0.001) and effector memory CD45RO^−^CD62L^−^ Tcyt cell frequencies (r = −0.38, *p* = 0.029). Moreover, a positive correlation with CD45RO^−^CD62L^+^ Th (r = 0.36, *p* = 0.040) and CD45RO^−^CD62L^+^ Tcyt cells (r = 0.60, *p* < 0.001) and TREC levels was observed. In addition, KREC concentrations in this group showed a positive correlation with relative and absolute numbers of CD19^+^ B cells (r = 0.38, *p* = 0.031 and r = 0.47, *p* = 0.006, respectively), as well as with the levels of CD19^+^CD5^+^ (r = 0.43, *p* = 0.012) and CD19^+^CD5^−^ cells (r = 0.35, *p* = 0.045) in peripheral blood.

## 4. Discussion

Patients in the acute stage of COVID-19 and COVID-19 survivors demonstrate specific alterations in circulating immune cell subsets [1,5,10,12]. The analysis of TREC and KREC levels characterized the functional activity of central organs of the immune system and, therefore, the release of naïve T and B cells into peripheral blood [13,14,16]. Previous studies demonstrated the clinical significance of TREC and KREC concentrations in the prognosis of COVID-19 severity. For instance, Khadzhieva et al. (2021) showed that TREC and KREC concentrations in COVID-19 patients with respiratory distress syndrome (RDS) were significantly lower than in patients without RDS [15]. Our study has also demonstrated the dependence of TREC and KREC levels on the course of COVID-19. In particular, in COVID-19 patients with a favorable disease outcome, blood TREC and KREC concentrations were significantly lower than the control values. The majority of COVID-19 patients with a poor outcome exhibited zero TREC levels (*n* = 7; 87.5%). At the same time, the KREC concentrations in this group were similar to the values observed in patients with a favorable disease outcome. In COVID-19 survivors, TREC and KREC concentrations were higher if compared to patients in the acute stage of the disease and were very closed to reference ranges. Thus, we can conclude that a low concentration of TREC during the acute stage of COVID-19 could predict an unfavorable outcome, whereas high levels of TREC and KREC indicated a favorable outcome of the disease.

As TREC and KREC concentrations are thought to be related to the formation of naïve T and B cells, respectively [13,14,15], we investigated the main subsets of peripheral blood lymphocytes in COVID-19 patients at different stages of the disease.

Firstly, T cells are involved in the key mechanisms of an antiviral immune response. Blood T cell counts decrease during the acute stage of COVID-19 [22,23]. A study by Diao et al. demonstrated that T cell levels correlated with IL-6 and IL-10 concentrations [9]. Our study has shown that absolute and relative numbers of T cells decreased in acute COVID-19. Furthermore, in patients with a favorable and a poor outcome, T cell levels decreased by 14.2% and 18.6%, respectively. COVID-19 survivors demonstrated an increase in T cell frequencies compared to healthy controls and patients during the acute disease period. However, the absolute T cell numbers in COVID-19 survivors were within the reference range but significantly higher than in patients during the acute stage of the disease: 3.1-fold and 3.5-fold compared to the patients with favorable and poor outcomes, respectively.

The assessment of main T cell subsets in COVID-19 patients at different stages of the disease showed that the lowest levels of Th cells (CD3^+^CD4^+^) were observed during the acute disease stage in patients with a poor outcome. For the patients who had a favorable outcome, T helper cells were within the reference range, while in COVID-19 survivors, this parameter was significantly increased (by 11.9% and 43.2% compared to the control values and patients with a poor disease outcome, respectively). At the same time, the percentages of cytotoxic T cells (CD3^+^CD8^+^) in patients during the acute stage of the disease and COVID-19 survivors were within the reference range. Changes in the absolute counts of T helpers and cytotoxic T cells in the blood in patients during the acute stage of the disease and COVID-19 survivors were equivalent to the changes in the total absolute T cell count, which was decreased during the acute stage of the disease and increased (including compared to the control values) after the recovery.

Effector T cell subsets are formed in two stages: in the thymus (antigen-independent differentiation) and in the peripheral organs of the immune system (antigen-dependent differentiation) [24]. CD45RO and CD62L expression could evaluate the T cell differentiation status. In naïve T cells (which have passed only the stage of antigen-independent differentiation), the extracellular protein of this receptor includes all domains (an antigen with a molecular weight of 220 kDa is defined as CD45RA) [25]. The transformation of CD45RA to CD45RO (with a molecular weight of 180 kDa) occurred as part of the alternative RNA splicing with antigen-dependent differentiation; therefore, CD45RO expression was observed in the central (CM) and effector (EM) memory T cells [26]. Terminally differentiated T lymphocytes (TEMRA) (cells at the final stage of their maturation) were also negative for CD45RO expression [27]. CD62L was another molecule that was expressed on T lymphocytes and determined their subset. CD62L (L-selectin, LECAM-1) was defined as a Ca^2+^-dependent C-like lectin involved in adhesion processes and acting as a “homing receptor” of lymphocytes in the peripheral lymphoid organs [28]. CD62L expression was observed on naïve and CM T cells. However, it is not expressed on EM and TEMRA T lymphocytes, as they function outside the lymphoid organs [29].

Therefore, the expression of these markers suggests that SARS-CoV-2 infection is associated with impaired development of effector T cell subsets. During the acute stage of the infection, patients demonstrated decreased blood counts of both TEMRA subsets (TEMRA T helpers—CD3^+^CD4^+^CD45RO^−^CD62L^−^; TEMRA cytotoxic T cells—CD3^+^CD8^+^CD45RO^−^CD62L^−^), i.e., the cells with the highest functional activity, which can be due to the migration of cells into the tissue to implement their function. However, in patients with an unfavorable outcome of this infectious process, the TEMRA T cell count is within the reference range (TEMRA T helpers) or significantly higher than the control values (TEMRA cytotoxic T cells). However, there is a decrease in the counts of naïve (CD3^+^CD4^+^CD45RO^−^CD62L^+^), CM (CD3^+^CD4^+^CD45RO^+^CD62L^+^) and EM cells (CD3^+^CD4^+^CD45RO^+^CD62L^−^); among the cytotoxic T cells, the counts of naïve (CD3^+^CD8^+^CD45RO^−^CD62L^+^) and CM cells (CD3^+^CD8^+^CD45RO^+^CD62L^+^) are decreased. This alteration of effector T cells might be related to the impaired mechanisms of cell maturation at the stage of antigen-independent differentiation (which is confirmed by the minimum blood TREC levels) and the mechanisms of the migration of effector cells into the tissue to implement their function. In particular, a study by Toor et al. showed that the detection of functionally active T cells with high levels of cytokine synthesis and perforin expression was associated with a more severe COVID-19 course [30]. COVID-19 survivors were found to have normal counts of effector T cells but lower levels of EM cytotoxic T cells. Furthermore, Gong et al. also demonstrated that COVID-19 survivors had normal levels of naïve CD4^+^ T cells (within the reference range), but the EM cell counts were found to be increased [31]. It can be concluded that at this stage of the infectious process, the antigen-independent differentiation of T cells was restored and followed by their distribution in the peripheral tissues. This assumption is supported by the fact that only COVID-19 survivors demonstrated correlations between the TREC concentrations and blood effector T cell counts. Moreover, positive correlations were revealed between the TREC levels and the naïve T cell counts, characterizing the restoration of the processes of T lymphocyte antigen-independent differentiation, whereas there were negative correlations between the TREC levels and the effector T cell counts (CM Th and TEMRA Tcyt).

B cells were also actively involved in the development of effective antiviral immune responses. Many studies have described disorders of B cell maturation and differentiation in COVID-19 patients resulting in decreased ‘naïve’ B cell and memory B cell blood counts [11,32,33]. Moreover, Golovkin et al. showed that severe COVID-19 was associated with an imbalance in memory B2-cell counts [23]. During the acute stage of COVID-19, both absolute counts and percentages of B cells decrease and so do almost all their subsets (except for naïve CD19^+^CD5^−^CD27^−^ cells). Furthermore, a decreased proportion of naïve IgD+CD38– B cells noted in patients with COVID-19 may point to altered bone marrow B cell differentiation [23]. Similarly, Kaneko et al. showed that patients with COVID-19 had lowered peripheral blood relative and absolute numbers of naïve IgD^+^CD27^−^ cells, transitional IgD^+^CD27^−^CD10^+^CD45RB^−^ and follicular CXCR5^+^ (IgD^+^CD27^−^CD10^−^CD73^+^) B cells compared with control subjects or convalescent patients [34]. These data indicated the decreased B cell bone marrow output.

During this period, patients demonstrated positive correlations between the KREC levels and blood CD5^+^ and CD5^−^ B cell counts. Therefore, it can be concluded that decreased B cell counts in the acute stage of COVID-19 are related to the impairment of their maturation at the level of antigen-independent differentiation. Furthermore, B cell subset alterations could persist for at least several months after the acute stage of the disease [35]. Such long-term impairment of adaptive humoral immunity may be closely associated with an increased risk of a wide range of autoimmune disorders [36,37], the incidence of which dramatically increases after COVID-19 [38].

The changes in B cell subsets in patients with a poor outcome of COVID-19 were quite different. These patients showed normal percentages of B cells in peripheral blood, while the B1-cell counts and the content of their subsets were increased. No changes in B2-cell subsets were demonstrated in patients with a poor outcome. Therefore, in COVID-19 with a poor disease outcome, B1-cell counts increase (due to naïve B1-cells and memory B1-cells), but B2 cell counts remain unchanged. It should be noted that these patients demonstrate no correlations between the KREC levels and the B cell subsets frequencies.

Finally, COVID-19 survivors also had decreased absolute and relative numbers of B cells, similar to the acute stage of the disease. Low B cell counts were observed in COVID-19 survivors when B1- and B2-cells were decreased. However, in contrast to the acute stage of the disease, COVID-19 survivors demonstrated restoration of naïve B cell counts (both naïve B1- and B2-cells). These results reflect the process of restoration of antigen-independent differentiation of B lymphocytes, which was confirmed by a significant increase in the blood KREC levels in this group of patients. In addition, COVID-19 survivors demonstrated positive correlations between the blood KREC levels and the total B cell count (as well as B1- and B2-cells).

## 5. Conclusions

The results indicate that TREC and KREC level alterations characterized the stage and the severity of COVID-19. Patients with a favorable disease outcome showed decreased levels of TREC and KREC in circulation during acute COVID-19 compared to healthy subjects. The lowest levels of TREC and KREC were observed in patients with a poor disease outcome. The highest levels of TREC and KREC were found in COVID-19 survivors. Based on the correlations between TREC and KREC concentrations and T and B cell subsets during the acute stage of COVID-19 and after recovery, it could be concluded that blood TREC and KREC levels could be sensitive indicators of antigen-independent differentiation of adaptive immunity cells. In general, the acute phase of COVID-19 in patients with a favorable outcome was characterized by decreased T and B cell blood counts. In these patients, T and B cell subset alterations included a significant decrease in TEMRA levels of Th cells and cytotoxic T cells, while there was a decrease in the B1- and B2-cell counts. In patients with a poor outcome of the acute stage of COVID-19, a decrease in the T cell count was associated with a decreased T helper count (due to insufficiency of naïve CM and EM cells), as well as a decreased cytotoxic T cell count (mainly due to naïve cells). An increase in the B cell count observed in patients with a poor disease outcome is associated with the B1-cell subsets. Blood T cell counts in COVID-19 survivors were higher than in healthy subjects (due to T helpers), while the effector subsets recovered almost completely. The qualitative and quantitative composition of blood B cell subsets in COVID-19 survivors was largely similar to that observed in the acute stage of the disease, while the counts of naïve B1- and B2-cell counts were restored. Thus, the change in the subset composition of T and B lymphocytes in patients with COVID-19 determines the nature of the course and outcome of the disease. The subset composition of T and B cells is not restored to control values in recovered patients, but a tendency to its normalization was revealed. Based on the revealed correlations between the levels of TREC and KREC concentrations with the subset composition of T and B lymphocytes as well as the information content of their levels in assessing the nature of the course and prognosis of the outcome, it can be concluded that it is necessary to determine the concentrations of TREC and KREC in the treatment of patients with COVID-19.

## Figures and Tables

**Table 1:** Clinical characteristics of groups of patients.

Characteristics	Acute Stage of COVID-19 (Favorable Outcome) *n* = 25	Acute Stage of COVID-19 (Unfavorable Outcome) *n* = 8	COVID-19 Survivors *n* = 33	*p*
Female	14/56.0%	6/75.0%	18/54.55%	*p*_1_ = 0.751 *p*_2_ = 1.000 *p*_3_ < 0.001
Male	11/44.0%	2/25.0%	15/45.45%	*p*_1_ = 0.700 *p*_2_ = 1.000 *p*_3_ = 0.707
Mean age	60 56.0–63.0	70 62.3–73.3	63 52.5–68.5	*p*_1_ = 0.989 *p*_2_ = 0.994 *p*_3_ = 0.875
Age group				
<45	3/12.0%	1/12.5%	5/15.15%	*p*_1_ = 1.000 *p*_2_ = 1.000 *p*_3_ = 1.000
45–59	15/60.0%	1/12.5%	18/54.55%	*p*_1_ = 0.238 *p*_2_ = 0.830 *p*_3_ = 0.249
≥ 60	7/28.0%	6/75.0%	10/30.3%	*p*_1_ = 0.171 *p*_2_ = 1.000 *p*_3_ = 0.183
Severity				
Moderate	7/28.0%	0/0%	16/48.48%	*p*_1_ = 0.309 *p*_2_ = 0.325 *p*_3_ = 0.090
Severe	17/68.0%	1/12.5%	17/51.52%	*p*_1_ = 0.012 *p*_2_ = 0.007 *p*_3_ = 0.413
Extremely Severe	1/4%	7/87.5%	0/0%	*p*_1_ < 0.001 *p*_2_ = 1.000 *p*_3_ = 0.001
Complications				
Pneumonia	22/88.0%	8/100%	32/96.67%	*p*_1_ = 0.989 *p*_2_ = 0.994 *p*_3_ = 0.875
Data of CT scan 3–4	1/4.0%	8/100%	0/0%	*p*_1_ < 0.001 *p*_2_ = 0.431 *p*_3_ < 0.001
Respiratory failure	12/48.0%	8/100%	14/42.42%	*p*_1_ = 0.355 *p*_2_ = 0.816 *p*_3_ = 0.224
Systemic inflammatory response syndrome	5/20.0%	6/75.0%	9/27.27%	*p*_1_ = 0.132 *p*_2_ = 0.765 *p*_3_ = 0.165
Multiple organ failure	2/8.0%	3/37.5%	4/12.1%	*p*_1_ = 0.078 *p*_2_ = 0.690 *p*_3_ = 0.120
Septic shock	0/0%	1/12,5%	0/0%	*p*_1_ = 0.242 *p*_2_ = 1.000 *p*_3_ = 0.195
Several complications	13/52.0%	8/100%	15/45.45%	*p*_1_ = 0.363 *p*_2_ = 0.819 *p*_3_ = 0.232
Concomitant disorders including				
Hypertension	10/40.0%	5	27/81.82%	*p*_1_ = 0.509 *p*_2_ = 0.131 *p*_3_ = 0.764
Diabetes	2/8.0%	3	5/15.15%	*p*_1_ = 0.134 *p*_2_ = 0.690 *p*_3_ = 0.355
Cardiovascular diseases	3/12.0%	4	8/24.24%	*p*_1_ = 0.168 *p*_2_ = 0.505 *p*_3_ = 0.434
Chronic kidney disease	0/0%	2/25.0%	1/3.03%	*p*_1_ = 0.076 *p*_2_ = 1.000 *p*_3_ = 0.125
Chronic pulmonary disease	1/4.0%	2/25.0%	2/6.06%	*p*_1_ = 0.181 *p*_2_ = 0.818 *p*_3_ = 0.209
Chronic liver disease	0/0%	2/25.0%	2/6.06%	*p*_1_ = 0.076 *p*_2_ = 0.506 *p*_3_ = 0.209
Other disorders	2/8.0%	2/25.0%	3/9.09%	*p*_1_ = 0.291 *p*_2_ = 1.000 *p*_3_ = 0.295
Several disorders	15/60.0%	8/100%	18/54.55%	*p*_1_ = 0.549 *p*_2_ = 0.830 *p*_3_ = 0.380
CT scan findings				
CT1	9/36.0%	0/0%	17/51.52%	*p*_1_ = 0.168 *p*_2_ = 0.631 *p*_3_ = 0.090
CT2	15/60.0%	0/0%	12/36.36%	*p*_1_ = 0.044 *p*_2_ = 0.353 *p*_3_ = 0.175
CT3	1/4.0%	1/12.5%	3/9.09%	*p*_1_ = 0.454 *p*_2_ = 0.633 *p*_3_ = 1.000
CT4	0/0%	7/87.5%	0/0%	*p*_1_ < 0.001 *p*_2_ = 1.000 *p*_3_ < 0.001

Note: *p*_1_—statistically significant differences between patients in the acute stage of COVID-19 with favorable and poor outcomes; *p*_2_—statistically significant differences between patients in the acute stage of COVID-19 with favorable outcomes and COVID-19 survivors; *p*_3_—statistically significant differences between patients in the acute stage of COVID-19 with poor outcome and COVID-19 survivors.

## Data Availability

Not applicable.
